# Protocol to study monocyte transmigration across primary human liver endothelial cells under physiological shear flow conditions *in vitro*

**DOI:** 10.1016/j.xpro.2024.103431

**Published:** 2024-11-05

**Authors:** Alex L. Wilkinson, Megan E. Bannister, Ayla O’Keeffe, Chris J. Weston, Patricia F. Lalor, Shishir Shetty, Daniel A. Patten

**Affiliations:** 1 Centre for Liver and Gastrointestinal Research, Institute of Immunology and Immunotherapy, University of Birmingham, B15 2TT Birmingham, UK; 2 National Institute for Health Research (NIHR), Birmingham Biomedical Research Centre, Birmingham, UK

**Keywords:** Cell isolation, Cell-based Assays, Immunology

## Abstract

Modeling immune cell recruitment by liver endothelial cells *in vitro* is important to better understand the pathology of chronic inflammatory liver diseases and cancers. Here, we present a protocol for the study of monocyte transmigration across activated primary human liver endothelial cells, under physiological flow conditions. We describe primary endothelial cell isolation from human liver tissues and monocyte isolation from human blood. We then detail the shear flow-based assay and subsequent analysis of the different stages of monocyte transmigration.

For complete details on the use and execution of this protocol, please refer to Wilkinson et al.^1^

## Highlights

• Step-by-step guide for isolating and culturing human liver endothelial cells
• Protocol for isolating monocytes from human blood
• Undertaking shear flow-based assays of monocytes across liver endothelial cells
• Quantitative analysis of monocyte transmigration route

## Abstract (editor highlights)

Publisher’s note: Undertaking any experimental protocol requires adherence to local institutional guidelines for laboratory safety and ethics.

## Graphical abstract

**Figure:**
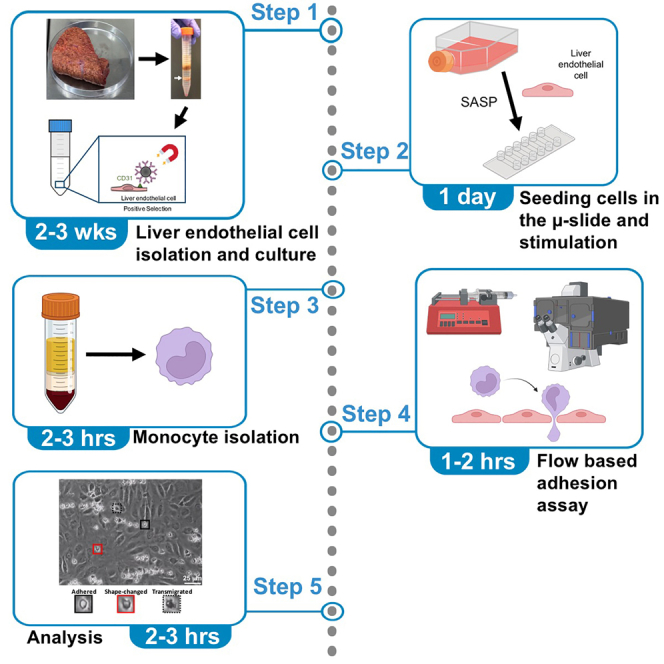

## Before you begin

The protocol below describes the specific steps for the isolation and culture of primary liver endothelial cells from human explanted and rejected donor liver tissues. Next, we describe the isolation of monocytes from healthy volunteer blood and the verification of their viability and purity. Finally, we describe the protocol for a shear flow-based adhesion assay to study the transmigration of monocytes across a monolayer of activated liver endothelial cells and subsequent analyses.

We would recommend that liver endothelial cells be isolated no later than 48 h after surgical retrieval, as tissue viability will be compromised. We have previously successfully isolated and cultured liver endothelial cells from patient tissues with common chronic liver disease etiologies, such as metabolic-associated steatohepatitis (MASH) and alcohol-related liver disease (ARLD), but also a wide variety of much rarer diseases, such as primary biliary cholangitis (PBC), primary sclerosing cholangitis (PSC) and Wilson’s disease. We have also previously shown that the isolated cells maintain their highly specialized endocytic phenotype in culture and differ significantly from more conventional vascular endothelial cells, such as human umbilical vascular endothelial cells (HUVECs), with a minimal basal membrane and loosely organized cell-cell junctions.^2^

Here, we use the proinflammatory senescence-associated secretory phenotype (SASP) collected from cells undergoing oncogene-induced senescence (OIS) and growing cell control supernatant^3^ to demonstrate how endothelial activation mediates the transmigration of monocytes. Nevertheless, a range of other inflammatory stimuli, such as TNF-α, could be utilized to induce endothelial activation.^4^

Monocytes should be isolated from blood collected in the presence of an anti-coagulant (we generally use EDTA blood collection tubes), to prevent clotting and the subsequent activation of monocytes. Here, we utilize healthy volunteer blood, but patient blood could also be used. We also recommend that monocyte purity be checked periodically by flow cytometry, to confirm that the monocyte isolation kit is performing as expected. Finally, the shear flow-based assay^5^ should be carried out immediately after the isolation of monocytes to limit their activation with prolonged storage.

### Institutional permissions

Appropriate approval from your local ethics committee is required to undertake experiments using patient-derived tissues or healthy volunteer blood required for this protocol. Human liver tissue and blood samples were collected with written informed consent and local ethics committee approval. Explant human liver tissue was collected from patients undergoing liver transplantation at the Queen Elizabeth Hospital Birmingham under ethical study numbers 06/Q2702/61, 18/WA/0214 and 18/LO/0102. Normal liver tissue was obtained from rejected donor organ deemed unsuitable for transplantation under LREC study numbers 06/Q2702/61 and 18/WA/0214. Peripheral blood samples were collected from healthy volunteers under LREC study number 18/WA/0214.

### Preparation of reagents

**Timing: 1 h**

1. Enzyme digestion stock (Collagenase I)∗.
  a. Sterile PBS was used to reconstitute Collagenase I to a working concentration of 10 mg/mL.

***Note:*** ∗Aliquots of this can be made in advance and stored at −20°C for up to 1 year to reduce the time spent preparing reagents when performing this protocol.

2. Liver endothelial cell medium.
  a. Described in key resources table.
3. Percoll dilutions.
  a. Prepare a working solution of stock Percoll with 99 mL Percoll and 11 mL 10x PBS.
  b. Prepare a 33% Percoll dilution of 33 mL Percoll and 67 mL 1x PBS.
  c. Prepare a 77% Percoll dilution of 77 mL Percoll and 23 mL 1x PBS.

***Note:*** Percoll stock and dilutions can be stored at 2°C–8°C for up to 6 months.

4. Reconstitute Fixable Viability Stain 620 LIVE DEAD Stain.
  a. Add 230 μL fresh cell culture-grade Dimethyl Sulfoxide (DMSO) and vortex.

***Note:*** Reconstituted LIVE DEAD Stain can be stored at −20°C for up to 1 year.

5. Prepare MACS Buffer.
  a. Described in key resources table.

### Preparation of equipment

**Timing: 0.5 h**

6. Switch on Class II Microbiological Safety Cabinet (MSC).
7. Preheat orbital shaker (37°C).
8. Switch on BD Bioscience LSRFortessa X-20.
  a. The laser configuration used for this protocol is as follows: 488 nm Blue, 640 nm Red, 405 nm Violet, 561 nm Yellow/Green, 355 nm Ultra Violet.

***Note:*** This is a suggested flow cytometer and configuration, the following protocol can be run on various cytometers and configurations depending on instrumentation and reagent availability.

***Note:*** Antibody concentrations used were determined by single color titration and with appropriate compensation applied. Positive staining was determined based on a matched isotype control.

9. Coat T25 and T75 tissue culture flasks with rat tail collagen. (refer to section materials and equipment for preparation).

## Key resources table

**Table:** 

REAGENT or RESOURCE	SOURCE	IDENTIFIER
**Antibodies**		

Anti-EpCAM antibody (HEA125) - 1:11 (4.55 μg/mL)	Progen	61004 (RRID AB_2920684)
CD14 APC anti-human antibody (TÜK4) - 1:100 (per 50,000 cells)	Miltenyi Biotec	130-113-705 (RRID AB_2726246)
Isotype control antibody mouse IG2a APC (S43.10) - 1:100 (per 50,000 cells)	Miltenyi Biotec	130-113-831 (RRID AB_2733441)
Fixable viability stain 620 - 1:100 (per 50,000 cells)	BD Biosciences	564996 (RRID AB_2869636)
Phalloidin AF633 - 1:40	Invitrogen	A22284
Silicon rhodamine (SiR)-actin live cell actin probe - 1:1,000 (1 μM)	Spirochrome	SC001

**Biological samples**		

Explanted human liver tissues	Queen Elizabeth Hospital, Birmingham, UK	LREC Approval 06/Q2702/61, 18/WA/0214 and 18/LO/0102, South Birmingham, Birmingham, UK.
Healthy volunteer blood	University of Birmingham, Birmingham, UK	LREC Approval 18/WA/0214, South Birmingham, Birmingham, UK.

**Chemicals, peptides, and recombinant proteins**		

BSA fraction V - 0.1%	Gibco	15260037
CellTracker Green (CMFDA) - 10 μM	Invitrogen	C2925
Collagenase from *Clostridium histolyticum*, type IA - 2 mg/mL	Sigma	C9891
EDTA 1 mM	Sigma	E0270
Endothelial cell serum-free medium	Gibco	11111044
Fetal bovine serum	Gibco	10500064
Human serum – 10%	TCS Biosciences	CS100-500
Lympholyte-H cell separation media	Cedarlane	CL5020
PBS (Ca^2+^/Mg^2+^ free)	Thermo Fisher Scientific	BR0014G
Penicillin-streptomycin-glutamine - 1X	Gibco	10378016
Percoll- 33% and 77%	Sigma	GE17-0891-01
PFA – 4% in PBS	Alfa Aesar	J61899
Collagen type I from rat tail - 1:100 (40 μg/mL)	Sigma	C3867
Recombinant human HGF - 100 μg/mL	PeproTech	100-39H
Recombinant human VEGF - 100 μg/mL	PeproTech	100-20
TrypLE express enzyme - 1X	Gibco	12605010

**Critical commercial assays**		

Pan monocyte isolation kit (human)	Miltenyi Biotec	130-096-537
Dynabeads CD31 endothelial cell	Thermo Fisher Scientific	11155D
Dynabeads goat anti-mouse IgG	Thermo Fisher Scientific	11033
Dynabeads CD45	Thermo Fisher Scientific	11153D

**Experimental models: Cell lines**		

Senescence-associated secretory phenotype (SASP) isolated from ER:HRas^G12V^ IMR90 cells	Dr Matthew Hoare, University of Cambridge	Cat# CCL-186

**Other**		

15 mL centrifuge tubes	Corning	352095
25 cm^2^ culture flasks	Corning	430639
5 mL syringes	BD Emerald	307731
50 mL centrifuge tubes	Corning	430829
6-well culture plates	Corning	3516
75 cm^2^ culture flasks	Corning	430641U
Agitation chamber	Cole-Parmer	N/A
Centrifuge	Henderson Biomedical	N/A
Class II microflow safety cabinet	Holten	LaminAir 1.8
DynaMag-15 separation magnet	Invitrogen	12301D
Electronic solenoid valve	Lee Products	N/A
Glass Luer lock syringe	Popper & Sons, Inc.	N/A
Hemocytometer	Neubauer	N/A
Harvard syringe pump	Harvard Apparatus	PHD2000
Humidified incubator	PHC	IncuSafe
ibidi ibiTreat μ-Slide VI 0.4	ibidi	80606
LS separation column	Miltenyi Biotec	130-042-401
MidiMACS separator	Miltenyi Biotec	130-042-302
Silicon tubing - large (2 mm inner diameter, 4 mm outer diameter)	Fisher Scientific	FB50855
Silicon tubing - small (1 mm inner diameter, 3 mm outer diameter)	Fisher Scientific	FB50853
Sterile Pasteur pipettes	Fisher	13469108
Sterile scalpels	Swann-Morton	05XX
Petri dish	Thermo Fisher Scientific	150468

## Materials and equipment

• Liver endothelial cell medium:

**Table:** 

Reagent	Final concentration
Endothelial Serum-free Medium	N/A
Human serum	10%
Penicillin/streptomycin and glutamine (PSG)	1%
Vascular endothelial growth factor (VEGF)	10 ng/mL
Hepatocyte growth factor (HGF)	10 g/mL

Stored at 4°C for up to 1 month.

• Shear flow-based assay medium: Endothelial basal media with 0.1% bovine serum albumin (BSA).

Stored at 4°C for up to 6 months.

• Magnetic-activated cell sorting (MACS) buffer:

**Table:** 

Reagent	Final concentration
PBS	N/A
Fetal bovine serum (FBS)	2%
Ethylenediaminetetraacetic acid (EDTA) (0.25 M)	1 mM

Stored at 4°C for up to 6 months.

• Rat Tail Collagen (RTC): dilute RTC stock to 1% with PBS to make a final working concentration of 220 μg/mL.
  ○ Add 3 mL or 5 mL RTC to a T25 or T75, respectively, and incubate in a hood at 21°C–23°C for 2 min.
  ○ Remove RTC and leave the flask to dry for approximately 1 h.

Diluted RTC stored at 4°C for up to 6 months.

***Note:*** RTC-coated flasks can be precoated and, if stored appropriately to maintain sterility as much as possible, are useable for up to 1 month.

## Step-by-step method details

### Isolation of liver endothelial cells

**Timing: 6 h**

**Figure 1:**
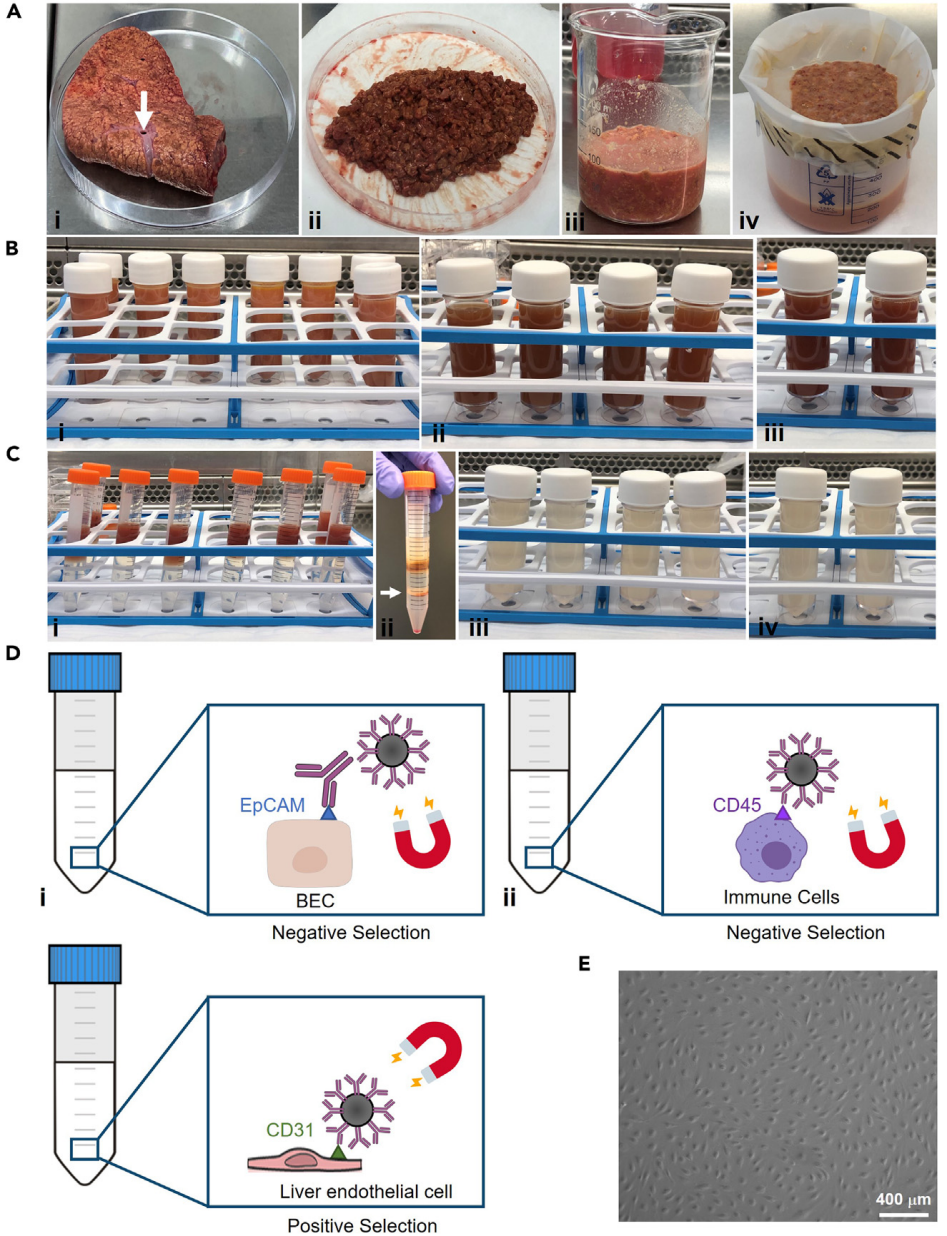
Isolation of primary liver endothelial cells from fresh human liver tissue (a diseased sample is shown) (A) (i) Liver slices from donor or diseased explants are (ii) mechanically chopped and (iii) enzymatically digested and (iv) strained through a fine mesh. (i) White arrow indicates a vascular area rich in connective tissue. (B) The digest is concentrated through a series of centrifugation steps to remove debris. (C) The digest is layered on top of a 33:77% Percoll gradient and (ii) the non-parenchymal cell fraction (white arrow) is isolated by density centrifugation. (iii and iv) Non-parenchymal cells are washed with phosphate buffered saline by centrifugation. (D) Biliary epithelial cells (BEC) and immune cells are removed by immunomagnetic selection against (i) epithelial cell adhesion molecule (EpCAM) and (ii) CD45, respectively. (iii) Liver endothelial cells are then isolated by positive immunomagnetic selection for CD31. (E) Cells are placed in culture in the presence of vascular endothelial growth factor and hepatocyte growth factor and passaged up to a maximum of five passages (p5). Scale bar represents 400 μm.

The following protocol describes the specific steps for the isolation of primary liver endothelial cells from explanted and rejected donor human liver tissues. The major steps are illustrated in Figure 1. For optimal results when isolating liver endothelial cells, human liver tissue samples should be placed in Dulbecco’s Modified Eagles Medium (DMEM) and stored at 4°C until processed.

***Note:*** Whilst some phenotypic changes will inevitably occur *in vitro*, we have previously demonstrated that primary human liver endothelial cells isolated using our protocol still maintain important phenotypic features, such as their capacity for rapid endocytosis via scavenger receptors and their unique junctional profile.^2^ Furthermore, they are phenotypically distinct from more conventional vascular endothelia, such as human umbilical vein endothelial cells (HUVECs).^2^ Liver endothelial cells were used up to passage 5.

**CRITICAL:** To maintain aseptic conditions, the following protocol needs to be carried out in a Class II Biological Safety Cabinet.

1. Digestion of liver tissue to a single cell suspension.
  a. Mechanically chop ∼75 g of human liver tissue with sterile scalpels in a 150 × 20 mm petri dish, until you reach the consistency of a course paste (Figure 1Aii).
    ***Note:*** Before chopping the liver, try to excise any highly vascular regions which will be rich in tough connective tissue, as indicated in Figure 1Ai.
  b. Enzymatically digest the liver tissue with collagenase I.
    i. Transfer the diced liver to a sterile glass beaker and add 20 mL of sterile PBS (Figure 1Aiii).
    ii. Add 5 mL of collagenase I (10 mg/mL in PBS) to the liver/PBS mixture, cover with foil and incubate at 37°C in an orbital shaker with constant agitation for 30–45 min.
      ***Note:*** We generally incubate normal liver with collagenase I for 30 min, but more cirrhotic tissues will require up to 45 min.
    iii. Strain the liver homogenate through a sterile beaker lined with fine mesh (Figure 1Aiv) to remove undigested tissue and debris.
2. Wash homogenate in PBS several times.
  a. Make volume of digest up to 200 mL with sterile PBS.
  b. Distribute the digest between 8 universal tubes, topping up with PBS as necessary.
  c. Centrifuge at 850 × *g* for 5 min at 21°C–23°C.
  d. Tip off the supernatant and resuspend the 8 pellets into 4 sterile universal containers (Figure 1Bii), washing out unused containers with PBS to ensure maximal retention of cells.
    ***Note:*** The pellets will be loose at this stage; therefore, care should be taken when discarding the supernatants to avoid unintentional loss of cells.
  e. Make up the volume to 25 mL with sterile PBS and centrifuge at 850 × *g* for 5 min at 21°C–23°C.
  f. Tip off supernatant and resuspend the 4 pellets into 2 sterile universal containers (Figure 1Biii), washing out unused containers with sterile PBS to again ensure maximal retention of cells.
  g. Make up the volume to 25 mL with PBS and centrifuge at 850 × *g* for 5 min at 21°C–23°C.
  h. Tip off supernatant and combine the pellets into once universal container and make up the volume with PBS to 24 mL.
3. Density gradient centrifugation to isolate non-parenchymal cells.
  a. Add 3 mL of 33% Percoll to 8 × 15 mL centrifuge tubes.
  b. Carefully layer 3 mL of 77% Percoll underneath the 33% solution.
    ***Note:*** Be careful not to mix the two solutions too much when layering the 77% Percoll underneath the 33% solution, as this will disrupt the density gradient. A distinct interface between the two layers should be apparent when holding the tube up to a light source.
  c. Carefully add 3 mL of liver homogenate on top of each of the 8 Percoll gradient tubes (Figure 1Ci).
  d. Centrifuge at 850 × *g* for 25 min at 21°C–23°C, brake 0.
    ***Note:*** Set the brake to 0 when utilizing density gradients to ensure that the gradient is maintained, and interphase is not disturbed as the centrifuge slows.
  e. Remove and discard the top layer (around the 6 mL mark).
  f. Collect the band of cells at the interphase of the Percoll gradients (around the 3 mL mark; Figure 1Cii) and combine 2 aliquots into 1 universal container (4 universals in total; Figure 1Ciii).
  g. Make the volume up to 25 mL with sterile PBS and mix well to prevent the formation of another gradient.
  h. Centrifuge at 850 × *g* for 5 min at 21°C–23°C.
  i. Remove supernatant and combine 2 pellets into 1 universal container (2 universals in total; Figure 1Civ). Make volume up to 25 mL with sterile PBS and centrifuge at 850 × *g* for 5 min.
  j. Tip off supernatant and combine pellet into a 15 mL centrifuge tube. Make the volume up to 14 mL with sterile PBS and centrifuge at 850 × *g* for 5 min at 21°C–23°C.
4. Depleting biliary epithelial cells (BEC) (Figure 1Di) and immune cells (Figure 1Dii).
  a. Discard the supernatant and resuspend the cell pellet in 500 μL of sterile PBS.
  b. Add 50 μL EpCAM antibody and incubate at 37°C for 30 min in an orbital shaker to provide constant agitation.
    ***Note:*** During this 30 min incubation period, put 50 mL of sterile PBS on ice in preparation for the following steps.
  c. Make the volume up to ∼10 mL with sterile room temperature (21°C–23°C) PBS, mix well and centrifuge at 850 × *g* for 5 min at 21°C–23°C.
  d. Tip off supernatant and resuspend the pellet in 500 μL ice-cold sterile PBS.
  e. Add 10 μL Goat Anti-Mouse IgG Dynabeads and 50 μL CD45 Dynabeads (ensuring that the beads are thoroughly resuspended prior to addition).
  f. Mix well and incubate for 30 min on ice with constant agitation.
    ***Note:*** An orbital shaker or benchtop rocker can be used for this purpose.
  g. Make up the volume to ∼6 mL with ice-cold PBS and mix well.
  h. Loosen lid of centrifuge tube and place in DynaMag-15 Separation Magnet for 2 min.
  i. With the 15 mL centrifuge tube in the magnet pipette off the supernatant and transfer to a second 15 mL centrifuge tube (do not discard).
  j. Place the second tube back in the magnet for 2 min and pipette off supernatant enriched for endothelial cells into a new sterile 15 mL centrifuge tube.
  k. Make the volume of endothelial-enriched supernatant up to 14 mL with ice-cold PBS and centrifuge 850 × *g* for 5 min at 4°C.
5. Positive selection of endothelial cells (Figure 1Diii).
  a. Discard supernatant and resuspend pellet in 500 μL ice-cold PBS.
  b. Add 10 μL fully re-suspended CD31 Dynabeads and incubate at on ice for 30 min with constant agitation.
    ***Note:*** An orbital shaker or benchtop rocker can be used for this purpose.
  c. Make up the volume to ∼6 mL with ice-cold PBS and mix well.
  d. Loosen lid of centrifuge tube and place in DynaMag-15 Separation Magnet for 2 min.
  e. With the 15 mL centrifuge tube in the magnet pipette off the supernatant and discard.
  f. Remove tube from the magnet and resuspend bead-bound cells in 5 mL of complete liver endothelial medium.
6. Seeding tissue culture flask.
  a. Transfer cell suspension into a rat tail collagen (RTC)-coated T25 culture flask.
  b. Place in a humidified incubator at 37°C with a 5% CO_2_ atmosphere.
  c. After 24 h replace the medium and return to incubator.

***Note:*** The yield of liver endothelial cells is highly variable and tissue dependent. Purity of isolation can be determined by immunocytochemical staining or flow cytometry. Phenotype can be determined by immunocytochemical staining and functionality can be confirmed by assays such as endocytic uptake.^6^

### Culture of liver endothelial cells

**Timing: 2–3 weeks**

The following steps describe the culturing of isolated primary liver endothelial cells. Alternatively, frozen stocks can be thawed before being cultured.

***Optional***/***Alternatively*** (if using frozen liver endothelial samples).

***Note:*** We usually thaw endothelial cells on Thursdays for them to be seeded for experiments the following week.

7. Thawing of frozen liver endothelial cells.
  a. Collect required samples from liquid nitrogen and thaw quickly in appropriate heat bath (water or bead at 37°C).
  b. Transfer sample to 15 mL centrifuge tube and make up the volume to 14 mL with PBS.
  c. Centrifuge at 850 × *g* for 5 min at 21°C–23°C.
  d. Remove supernatant and resuspend the pellet in liver endothelial media.
  e. Transfer to RTC-coated T75 and place in humidified incubator at 37°C with a 5% CO_2_ atmosphere.
  f. After 24 h, replace the medium.
8. Passaging liver endothelial cells.
  a. Once cells have reached 80%–90% confluency, remove the medium and wash with PBS.
  b. Add 2 mL TrypLE Express Enzyme (1X) to the T25 flask.
    ***Note:*** Time taken to reach 80%–90% confluency is subject to isolation yield and donor variability.
  c. Incubate for 3–5 min, checking cells are no longer adhered.
  d. Remove the cell suspension and place in a 15 mL centrifuge tube.
  e. Make up the volume to 14 mL with PBS and centrifuge at 850 × *g* for 5 min at 21°C–23°C.
  f. Remove the supernatant and resuspend the pellet in liver endothelial medium at a cell density of 1 × 10^6^ cells/mL.
  g. Transfer approx. 5 × 10^5^ cells to an RTC-coated T75 tissue culture flask and add 10 mL of liver endothelial medium.
  h. Place in a humidified incubator at 37°C with a 5% CO_2_ atmosphere.
  i. Replace medium every 2–3 days, until 80%–90% confluence is reached.
    ***Note:*** Liver endothelial cells were cultured in this study to passage 3 (p3), at which they were seeded into an ibiTreat μ-slide VI 0.4.

### Seeding and stimulating liver endothelial cells in the flow channel μ-slide

**Timing: 1 day**

This step describes the seeding of liver endothelial cells in commercially available flow chamber slides and the treatment of the monolayer to activate them and induce the expression of adhesion molecules and chemokines etc. which are key to the transmigration of monocytes.

9. μ-Slide preparation.
  a. Pipette 100 μL RTC solution into each lane of an ibiTreat μ-Slide VI 0.4 (Ibidi) and incubate for 30 min at 37°C.
  b. Wash the channels through twice with sterile PBS and leave the second wash in the μ-slide to prevent the μ-slide drying out.
10. Seeding cells in μ-slide.
  a. Once cells have reached 80%–90% confluency, remove the medium and add 3.5 mL TrypLE Express Enzyme (1X) to the T75 flask.
  b. Incubate for 3–5 min, checking cells are no longer adhered.
  c. Remove the cell suspension and place in a 15 mL centrifuge tube.
  d. Make up the volume to 14 mL with PBS and centrifuge at 850 × *g* for 5 min at 21°C–23°C.
  e. Remove the supernatant and resuspend the pellet in liver endothelial medium at 1 × 10^6^ cells/mL.
  f. Pipette 75 μL (75,000 cells) of cell suspension directly into each channel of the μ-Slide chamber.
  g. Leave cells to adhere for 1 h in a humidified incubator at 37°C with a 5% CO_2_ atmosphere.
  h. Fill the channels either slide with complete liver endothelial medium and return to the incubator for 24 h.
11. Stimulation of cells.
  ***Note:*** Stimulation should be performed 24 h prior to shear flow-based assay.
  a. Replace liver endothelial medium with 3 × 100 μL of endothelial medium containing activating agent.∗
    **CRITICAL:** When removing culture medium from Ibidi μ-slide lanes, we recommend leaving a small volume of medium in each channel to avoid damaging the liver endothelial monolayers. Change the culture medium by pipetting from one port and pipetting into the opposite port to wash new medium through the channel. Repeating this wash through 2–3 times will ensure thorough medium exchange.
    ***Note:*** Here, we utilize the senescence-associated secretory phenotype (SASP) collected from cells undergoing oncogene-induced senescence (ER:HRas^G12V^ IMR90 cells) and growing cell control supernatants.^3^ At this stage of the protocol, liver endothelial monolayers can be exposed to various treatments such as cytokines (e.g. TNF-α and IFN-γ;^2^), chemokines, microbial antigens (e.g. LPS^4^), ionizing radiation^7^ etc. to induce endothelial activation.
  b. Incubate in humidified incubator at 37°C with a 5% CO_2_ atmosphere for 24 h.
    ***Note:*** Prior to this step, siRNA knockdown can be utilized to induce the transient silencing of genes which encode for proteins of interest. We recommend performing the siRNA knockdown, then letting the endothelial cells equilibrate in antibiotic- and growth factor-free medium for 24 h, then treating for 24 h to induce endothelial activation.^1^^,^^4^^,^^8^

### Monocyte isolation

**Timing: 2–3 h**

This section describes the negative isolation of CD14^+^ monocytes from healthy volunteer blood. We use negative selection to avoid surface-bound antibodies on our target cells which may interfere with the flow-based adhesion assays.

**Figure 2:**
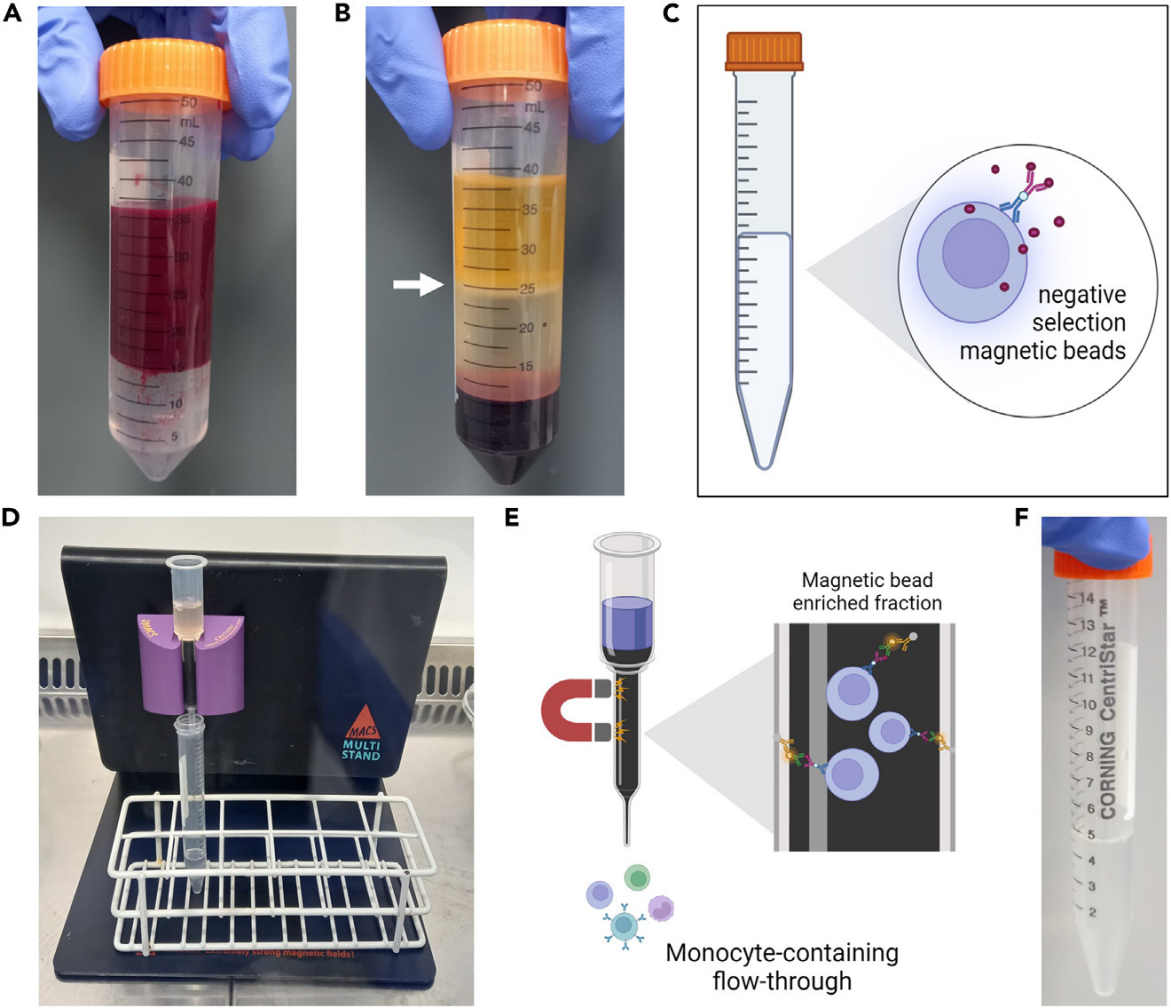
Isolation of monocytes from fresh human blood (A) Whole blood is carefully layered on top of Lympholyte-H Cell Separation Media. (B) The buffy layer (white arrow), containing peripheral blood mononuclear cells (PBMCs), is collected, transferred to a new 15 mL and PBMCs are washed in MACS buffer and counted. (C) Non-monocytes are labeled with the antibody cocktail and magnetic beads included in the Pan Monocyte isolation kit (Human; Miltenyi) (D and E) Monocytes are negatively selected out of the PBMC suspension by passing the PBMC suspension through an LS Separation column in the magnetic field of a MidiMACS separator. (F) Purified monocytes are collected and counted before being used in the shear flow-based assay.
  c. Centrifuge at 850 × *g* for 20 min, with no brake at 21°C–23°C.
13. Isolation of buffy coat layer.
  a. Remove the buffy layer containing peripheral blood mononuclear cells (PBMCs; Figure 2B) and transfer to a sterile 50 mL centrifuge tube.
  b. Make up the volume to 40 mL with sterile PBS and centrifuge at 850 × *g* for 5 min 21°C–23°C.
  c. Discard supernatant and resuspend pellet in 40 mL sterile PBS. Centrifuge at 350 × *g* for 10 min at 21°C–23°C to remove platelets.
  d. Discard the supernatant and resuspend pellet in 20 mL sterile PBS.
  e. Pass the PBMC suspension through a 70 μm cell strainer to remove any clumps and to achieve a single cell suspension.

***Note:*** The following steps should be performed as quickly as possible, and all solutions should be pre-cooled on ice. This helps to prevent the activation of monocytes.

***Note:*** Volumes given below are for up to 10^7^ total PBMCs. When working with fewer cells, use the same volumes as indicated. When working with higher cell numbers, scale up volumes accordingly.

12. Density gradient centrifugation of whole blood to obtain PBMC fraction.
  a. Add 15 mL of Lympholyte-H Cell Separation Media to a 50 mL centrifuge tube.
  b. Carefully layer 20–30 mL of whole blood on top of the Lympholyte-H (Figure 2A).

14. Cell number determination and magnetic labeling using the Pan Monocyte Isolation Kit (Human; Miltenyi) (Figure 2C).
  a. Determine the total number of PBMCs using a hemocytometer.
  b. Centrifuge the PBMC suspension at 850 × *g* for 5 min at 21°C–23°C and discard supernatant.
  c. Resuspend cell pellet in 30 μL of MACS buffer per 10^7^ total PBMCs.
  d. Add 10 μL of FcR Blocking Reagent per 10^7^ total PBMCs.
  e. Add 10 μL of Biotin-Antibody Cocktail per 10^7^ total PBMCs.
  f. Mix well (by pipetting up and down several times) and incubate for 5 min on ice with constant agitation.
    ***Note:*** An orbital shaker or on a benchtop rocker can be used for this purpose.
  g. Add 30 μL of buffer provided with the isolation kit per 10^7^ total cells.
  h. Add 20 μL of Anti-Biotin MicroBeads per 10^7^ total cells.
  i. Mix well and incubate for 10 min on ice with constant agitation.
    ***Note:*** An orbital shaker or on a benchtop rocker can be used for this purpose.
    ***Note:*** Whilst the PBMC suspension is incubating on ice, proceed to step 15 to prepare the LS separation column.
15. Magnetic cell separation.
  a. Place the LS Separation column in the magnetic field of MidiMACS separator.
  b. Prepare the LS column by rinsing with 3 mL MACS buffer and letting it run into a 15 mL centrifuge tube.
    ***Note:*** Always wait until the column reservoir is empty before proceeding to the next step.
  c. Apply the PBMC suspension to the column and collect flow through containing unlabeled cells (representing the enriched CD14^+^ monocytes) into a new sterile 15 mL centrifuge tube (Figures 2D and 2E).
  d. Wash the column with a further 3 mL of MACS buffer, collecting the flow through in the same collection tube used in the previous step (Figure 2F).
    ***Optional:*** Repeat steps 15a–15d.
16. Cell number determination and resuspension in shear flow-based assay medium.
  a. Determine the total number of monocytes using a hemocytometer.
  b. Centrifuge the monocyte suspension at 850 × *g* for 5 min and discard supernatant.
  c. Resuspend cell pellet in 1 mL of shear flow-based assay buffer (Endothelial SFM + 0.1% BSA) per 10^6^ total monocytes.

**CRITICAL:** After isolation, counting and resuspension in shear flow-based assay buffer, keep isolated monocytes on ice until required. This will limit monocyte activation before they enter the shear flow-based assay set-up and reduce the incidence of ‘clumping’ during the assay.

### Monocyte viability and purity

**Timing: 1–2 h**

This section describes the fluorescent staining of isolated CD14^+^ monocytes from healthy volunteer blood for flow cytometry analysis. Isolated monocytes are stained with fluorescent conjugated antibodies to determine their purity and viability via flow cytometry. Forward scatter and side scatter, along with the antibodies detailed in this section, allow the identification of viable monocyte populations.

***Note:*** Viability/purity analysis can be run periodically and in retrospect of the flow adhesion assay to confirm that the monocyte isolation kit is performing as expected as per the manufacturer’s description. We recommend that this is undertaken every 3–4 months or once per experiment series (whichever comes first).

17. Fluorescent staining of isolated monocytes.
  a. Add 1 μL Fixable Viability Stain 620 (FVS620) to ∼50,000 monocytes in 100 μL of ice-cold MACS buffer and incubate for 30 min on ice in the dark.
  b. Add 1 mL ice-cold MACS buffer and centrifuge cell suspension at 850 × *g* for 5 min.
  c. Remove the supernatant and resuspend the pellet in 100 μL ice-cold MACS buffer.
  d. Add 1 μL CD14-APC, or corresponding isotype control, to the 100 μL cell suspension.
  e. Incubate for 1 h on ice in the dark.
  f. Add 1 mL ice-cold MACS buffer and centrifuge at 850 × *g* for 5 min.
  g. Remove the supernatant.
  h. Resuspend the pellet in 250 μL ice-cold MACS buffer for acquisition on the cytometer.
18. Acquire data using an appropriate flow cytometer.

***Note:*** Here we used a BD Bioscience LSRFortessa X-20, but other machines can be used (see preparation of equipment for laser configuration).

**Figure 3:**
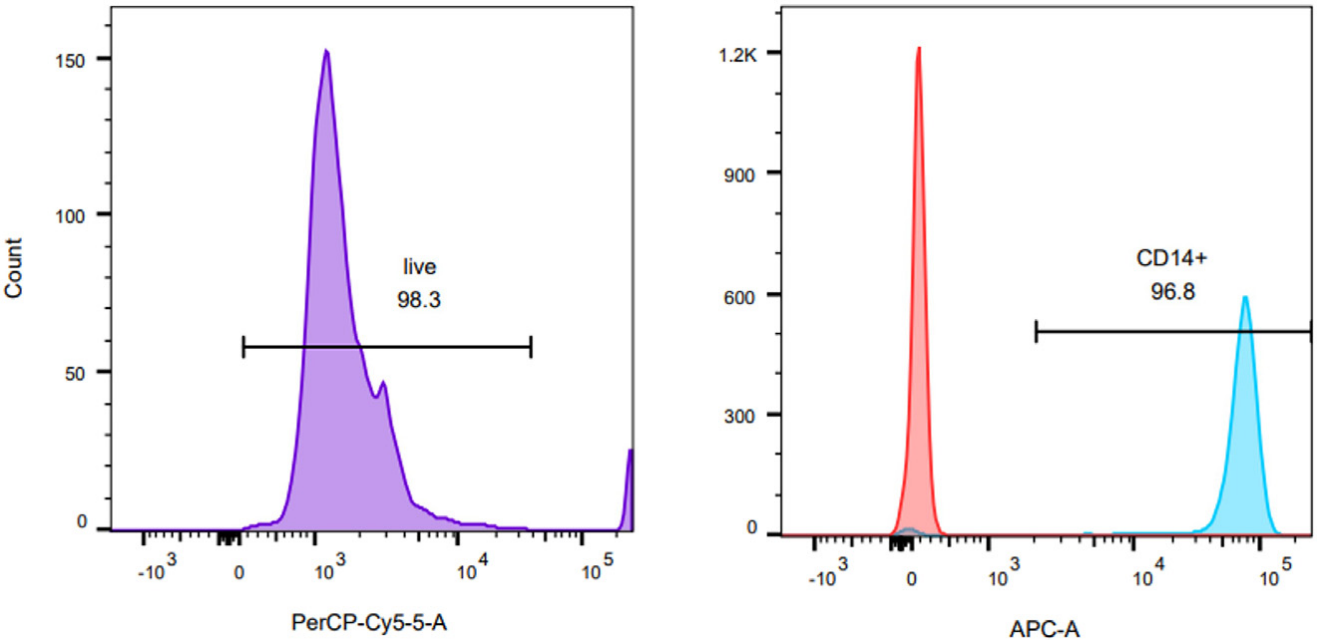
Evaluation of monocyte viability and purity Flow cytometry histograms of percentage live monocytes (left) and CD14^+^ monocytes (right). Red peak represents the isotype control.

19. Analyze flow cytometry data using appropriate software, such as FlowJo v.10.10.0 (Figure 3).

### Shear flow-based assay

**Timing: 1–2 h**

The shear flow-based assays were performed to recapitulate leukocyte recruitment within the hepatic sinusoids.^5^ For full description and visual demonstration of the shear flow-based assay system set up refer to Shetty et al.^5^; however, we describe the major steps below.

20. Set up of shear flow-based assay system.
  ***Note:*** The thermostatically controlled transparent chamber should be prewarmed to 37°C.
  a. Prefill a 50 mL glass Luer lock syringe with 10 mL of sterile distilled water.
  b. Attach a 25 cm length of small silicone tubing to the syringe port.
  c. Load the syringe into a syringe pump.
  d. Alter the rate of withdrawal (refill rate) according to the microslide manufacturer’s instructions and diameter of syringe to maintain a shear stress of 0.05 Pa (0.5 dyne/cm^2^).
    ***Note:*** For a μ-Slide VI 0.4 the refill rate is set to 0.28 mL/min to achieve a shear stress of 0.05 Pa in a tube of that specific dimensions.
  e. Attach two 5 mL syringes to the two in-flow ports of an electronic solenoid valve using a combination of 1 cm lengths of both large and small silicone tubing.
    ***Note:*** The electronic solenoid valve allows alternation between shear flow-based assay buffer and the monocyte suspension into the connected microslide.
  f. Attach a 12 cm length of small silicone tubing to the outflow port of the electronic solenoid valve.
  g. Insert shear flow-based assay buffer into both syringe barrels.
    ***Note:*** Ensure that the buffer is flowing from both barrels through the electronic solenoid valve to the out-flow silicone tubing.
  h. Alternate the valve switch to ensure flow and all bubbles are removed.
  i. Remove the shear flow-based assay buffer from the righthand barrel.
  j. Replace with monocyte suspension.
  k. Attach the microslide to the microscope stage with adhesive tape.
  l. Connect the first channel of the μ-Slide VI 0.4 and the syringe pump via a microslide adaptor (supplied by ibidi).
  m. Connect the opposite port to the outflow valve via a microslide adaptor.
    ***Note:*** Ensure the silicone tubing and adaptors are filled with shear flow-based assay buffer before μ-Slide VI 0.4 connection to prevent air bubbles forming.
  n. Set the microscope to 10X objective and the appropriate phase setting.
  o. Focus the microscope on the endothelial layer using the ocular lens.
  p. Ensure a camera attached to the microscope can relay images to a monitor and that you can record outcomes.
21. Shear flow-based assay technique.
  a. Perfuse the endothelial layer with shear flow-based assay buffer for 30 s.
    ***Note:*** This will remove any cell debris etc.
  b. Switch the valve to perfuse with monocyte suspension for 5 min at a shear stress of 0.05 Pa.
  c. Switch to perfuse the channel with shear flow-based assay buffer for 3 min.
  d. Record 10–12 fields of view along the length of the μ-Slide channel, recording for ∼10 s per field of view.
    ***Note:*** Ensure the recordings are made against the direction of flow to prevent recording any rolling cells twice.
  e. Save recordings and analyze “offline”.

### Monocyte transmigration quantification

**Timing: 2–3 h**

22. Open the saved image series in ImageJ.

***Note:*** Image J is freely downloaded from https://imagej.Nih.gov/ij/download.html (Schindelin et al.^9^).

23. Open the “Cell Counting” plugin.
24. Prepare an excel file for the resultant data to be inputted in to.

**Figure 4:**
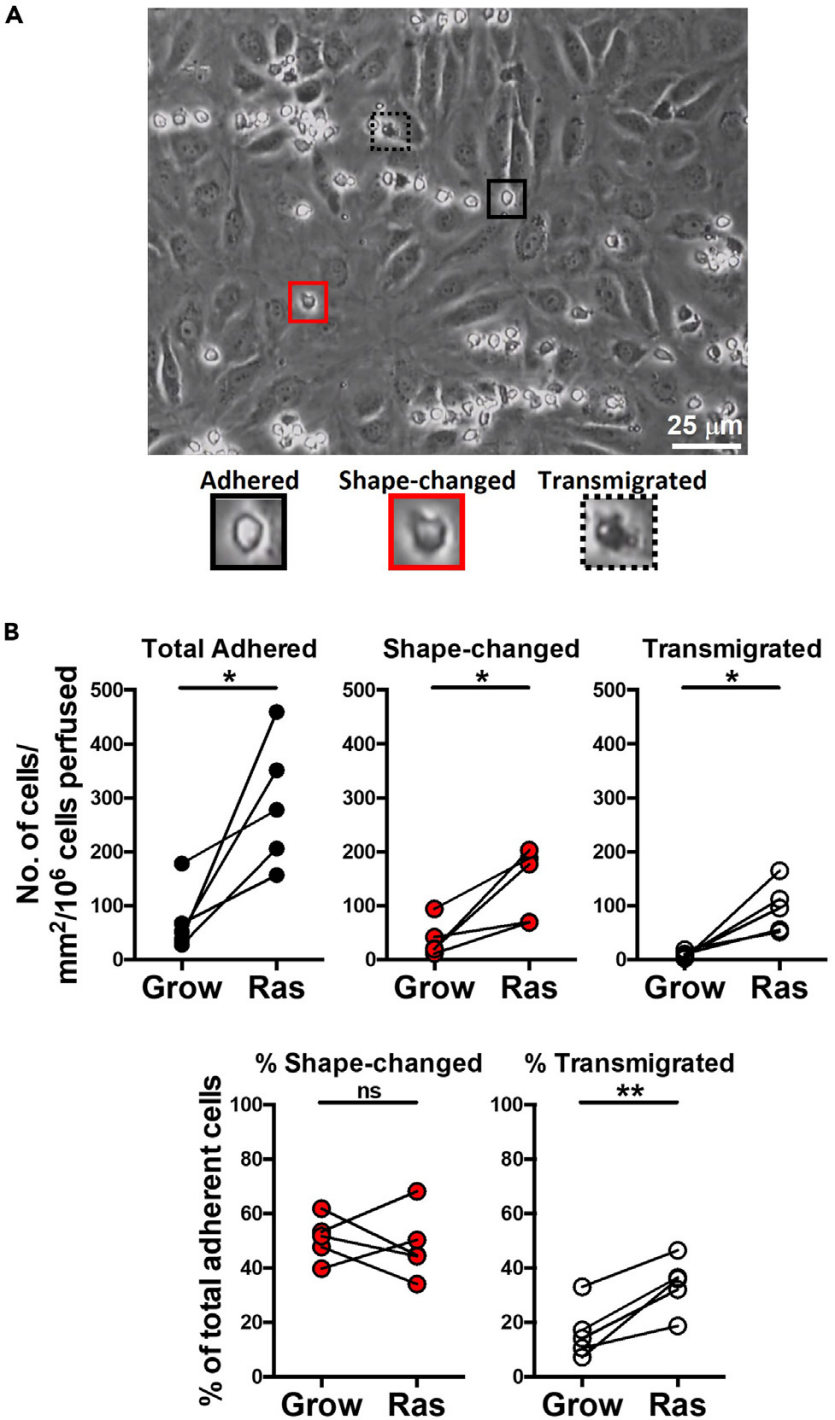
Identification of transmigration stage of monocytes across primary human liver endothelial cells Primary human liver endothelial cells are stimulated for 24 h with the senescence-associated secretory phenotype (SASP) collected from cells undergoing oncogene-induced senescence (ER:HRas^G12V^ IMR90 cells; Ras) and growing cell control supernatants (Grow). (A) Identifying adhered (*black square*), shape-changed (*red square*) and transmigrated (*dotted square*) monocytes via their morphology and phase brightness/darkness. Scale bar represents 25 μm. (B) Mean counts per lane are normalized to cells/mm^2^/10^6^ cells perfused (*upper panel*) and shape-changed and transmigrated monocyte numbers are expressed as a percentage of total adherent cells (*lower panel*). Here, we show that primary human liver endothelial cells activated with Ras supernatant support significantly higher levels of monocyte adherence and transmigration, when compared to controls treated with Grow supernatant. ∗ and ∗∗ indicate statistical significance, where *p* ≤ 0.05 or *p* ≤ 0.01, respectively. ns = not significant.

***Note:*** Ten fields of view are analyzed for the number of adhered, shape-changed and transmigrated monocytes per channel of the μ-Slide.

***Note:*** Stable, round, phase-bright monocytes are counted as “adhered” (Figure 4A), whilst monocytes that were phase-dark were considered “transmigrated” (Figure 4A). Shape-changed cells were no longer round and could be partially phase-bright/phase-dark (Figure 4A).

25. Mean cell counts per channel are normalized to cells/mm^2^/10^6^ cells perfused using the following equation:

$$N=\frac{c}{r\times b\times a\times l\;}$$

where N is the normalized count, c is the cell count per visual field, r is the flow rate (0.28 mL/min), b is the bolus time (i.e., 5 min), a is the visual field area (0.154 mm^2^ calculated by measuring the diameter of the field of view with a graticule and determining the total area with the formula for a circle: π × (0.5 × diameter)^2^) and l is the leukocyte concentration (1 × 10^6^/mL).

26. The proportion of monocytes undergoing activation (shape-changed) and transendothelial migration are expressed as a percentage of the total adherent cells (Figure 4B).

### Analysis of route of transmigration

**Timing: 5–6 h**

**Figure 5:**
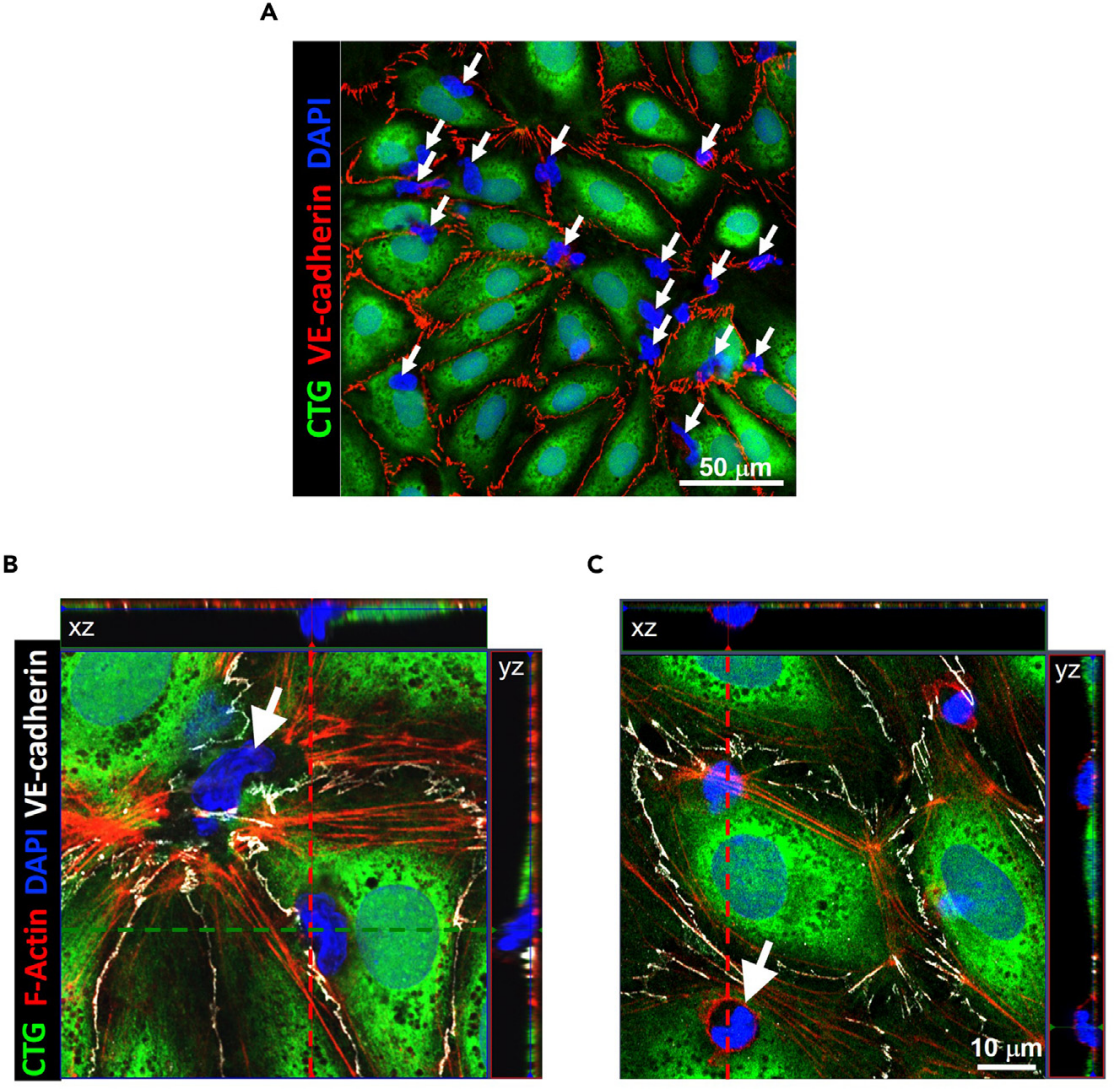
Identification of monocyte transmigration route across primary human liver endothelial cells (A) Monocytes (blue; white arrows) adhered to and migrating across a monolayer of liver endothelial cells (green). Endothelial cell-cell junctions are delineated with immunofluorescent staining of VE-cadherin (red). Scale bar represents 50 μm. (B) Transmigrating monocyte (blue; white arrow) utilizing the paracellular pathway between liver endothelial cells (green) causes a visible break in the VE-cadherin (white). (C) Example of a lymphocyte (blue; white arrow) transmigrating across a liver endothelial cell monolayer (green) via the transcellular pathway, which is characterized by an enrichment of F-actin (red). Scale bar represents 10 μm.

It is also possible to determine whether the migrating cells move through cell junctions (paracellular) or across the main body of the cell (transcellular) using fluorescence microscopy. To characterize the route of monocyte transmigration, liver endothelial cells can be pre-labeled with fluorescent dyes (CellTracker Green and SiR-actin; 30 min in shear flow-based assay medium) before shear flow-based adhesion assays with peripheral blood monocytes. These cells can then be fixed with 4% paraformaldehyde (PFA) and cell junctions can be subsequently visualized using immunofluorescent staining and confocal microscopy (Figure 5).

27. Transmigration events are first identified by the presence of a monocyte.

***Note:*** These can be visualized with DAPI and distinguished from liver endothelial cell nuclei based on size; Figure 5A), along with disruption of the liver endothelial cell cytoplasm (CellTracker Green; CTG), which allows differentiation from monocytes adhered to the endothelial surface.

28. The route of transmigration is determined by the location of diapedesis and the integrity of the VE-cadherin cell junctions.

***Note:*** disruption of VE-cadherin indicates a paracellular transmigratory event (Figure 5B).

29. Visualization of the liver endothelial cell cytoskeleton allows identification of immune cells undergoing transcellular transmigration (Figure 5C).

***Note:*** Pre-labeling the liver endothelial cells with the live-cell actin probe, SiR-actin allows the visualization of immune cells (largely lymphocytes) which transmigrate via the transcellular pathway, with an enrichment of F-actin evident around the immune cell (Figure 5C).

## Expected outcomes

### Liver endothelial cell isolation

Numbers of viable liver endothelial cells varies widely between liver tissue donors; however, we generally expect that isolated cells will be ready to culture within the first two weeks post-isolation. Once cultured into ibidi μ-Slides, viability should remain greater than 95%. If cell monolayers do not look intact, they should not be used for shear flow-based assays.

### Monocyte isolation from blood

We generally expect a >95% purity of CD14^+^ monocytes.

### Flow-based adhesion assay

In our experience, the total number of monocytes adhering to activated liver endothelial cells is highly variable and can range from 150 to 500 monocytes/mm^2^/10^6^ cells perfused, even within the same treatment group. This variability is likely due to the heterogeneity of patient tissues utilized in our isolation of liver endothelial cells, and the method of inducing activation of the liver endothelial cells, with different stimuli regulating the molecules involved monocyte transmigration to different degrees. Nevertheless, we generally observe 30%–60% of total adhered monocytes undergo transmigration across the liver endothelial monolayer (Figure 2B). We, and others, have shown that monocytes predominantly transmigrate via the paracellular pathway, with up to 90% of all transmigrating monocytes utilizing this pathway.^1^^,^^10^

## Limitations

Whilst we have previously shown that liver endothelial cells retain their phenotype and functionality in culture, thus maintaining a level of physiological relevance,^2^^,^^6^ this 2D *in vitro* assay is a very reductionist system in which monocytes interact in isolation with them. Recent advances in intravital microscopy mean that monocyte recruitment can now be elegantly imaged in “real-time” in injured murine liver tissues *in vivo*^11^; however, researchers must be mindful of potential species differences in endothelial properties when undertaking studies of that nature. Therefore, this system offers the ability to identify factors influencing transendothelial migration of human monocytes under physiological shear flow and can also be developed to incorporate laser scanning confocal microscopy for more detailed real-time imaging.^2^ In addition, pre-labeling with fluorescent dyes and subsequent immunofluorescent staining can be utilized for a more detailed analysis of monocyte adhesion and route of transendothelial migration (Figure 5). Nevertheless, the major drawback of a system of this nature is that the post-transmigration behaviors, such as interactions with other liver resident cell types and extracellular matrices, of liver-infiltrating monocytes cannot be observed, as with intravital imaging.

## Troubleshooting

### Problem 1

Lack of access to human liver tissues from which to isolate liver endothelial cells (steps 1–6).

### Potential solution

Liver endothelial cells are commercially available but advise phenotype check if used.

### Problem 2

Low yield of liver endothelial cells (steps 1–6).

### Potential solution

The yield of liver endothelial cells is highly variable and tissue dependent. For more cirrhotic samples, we extend the incubation period of the tissue homogenate with collagenase I to 45 min, to maximize tissue digestion and the release of cells.

### Problem 3

Contamination of liver endothelial cells isolates with monocytes/macrophages (step 6).

### Potential solution

In our experience, contamination with monocytes/macrophages is a common issue when isolating liver endothelial cells from rejected donor liver tissues. The contaminating monocytes/macrophages internalize the CD31 Dynabeads and are consequently carried into the resultant culture during the positive selection of endothelial cells. Nevertheless, they can be removed from endothelial cell cultures when splitting by placing the resultant cell suspension in DynaMag-15 Separation Magnet for 2 min. Endothelial cells lose the magnetic beads on their surface over time, but the internalized beads within the monocytes/macrophages mean that they will be positively selected out of suspension by the magnetic field (i.e., sticking to the side of the centrifuge tube) and the endothelial cells will remain in suspension. The endothelial cell suspension can then be removed by carefully pipetting out, taking care not to disturb the monocytes/macrophages on the side of the tube.

### Problem 4

Lack of access to healthy volunteer blood from which to isolate monocytes (steps 12–16).

### Potential solution

Whole blood and buffy coat layers are commercially available.

### Problem 5

Air bubbles forming in the shear flow-based assay set-up (steps 20–21).

### Potential solution

It is essential to prevent the formation of air bubbles within the shear flow-based assay set-up as they can damage the endothelial monolayer within the microslide or strip adherent monocytes from the endothelial surface. This can be avoided by ensuring all tubing and adaptors are thoroughly perfused with pre-warmed shear flow-based assay buffer before use. In addition, when connecting the microslide to the shear flow-based assay set-up, it is imperative to ensure that there is liquid/liquid interface between the adaptor and microslide port to avoid the introduction of air into the system.

### Problem 6

Monocytes clumping in the shear flow-based assay system (steps 20–21).

### Potential solution

Keeping monocytes on ice until immediately before being used in the shear flow-based assay will minimize their activation and clumping. In addition, passing the monocyte suspension through a 70 μm cell strainer will remove any large clumps and/or separate out clumping cells back into a single-cell suspension.

### Problem 7

Difficulty determining the stage of transmigration of monocytes (step 22–26).

### Potential solution

During recording, it is important to ensure that the endothelial layer is adequately in focus to allow accurate offline analysis. In addition, whilst recording a field view, we recommend toggling the plane of focus up and down before moving to the next field of view, as that can sometimes clarify where the monocyte sits relative to the endothelial monolayer.

## Resource availability

### Lead contact

Further information and requests for resources and reagents should be directed to and will be fulfilled by the lead contact, Prof Shishir Shetty, s.shetty@bham.ac.uk.

### Technical contact

Further information and technical help should be directed and fulfilled by the technical contact, Dr Daniel Patten, d.a.patten@bham.ac.uk.

### Materials availability

This study did not generate new unique materials.

### Data and code availability

The data used in support of the generation and optimization of this study have not been deposited in a public repository but are available from the corresponding author upon reasonable request.

## Acknowledgments

A.L.W. is funded by a Wellcome Trust PhD studentship in Mechanisms of Inflammatory Disease and a follow-on fund awarded by the University of Birmingham. M.E.B. is funded by an Engineering and Physical Sciences Research Council lifETIME CDT PhD studentship. A.O., S.S., and D.A.P. are funded by a Cancer Research UK Advanced Clinician Scientist Fellowship (C53575/A29959) awarded to S.S. This research is funded by the National Institute for Health and Care Research (NIHR) Birmingham Biomedical Research Centre (BRC). The views expressed are those of the author(s) and not necessarily those of the NIHR or the Department of Health and Social Care.

We thank Dr Matthew Hoare and Dr Kelvin Yin, University of Cambridge, for their kind gift of the senescence-associated secretory phenotype (SASP) supernatants (Ras) and growing cell supernatant controls (Grow) from ER:HRas^G12V^ IMR90 cells.

We also thank the patients and clinical staff from the Queen Elizabeth Hospital, Birmingham, for donation and collection of tissue and blood samples.

## Author contributions

A.L.W., data acquisition, data analysis, and figure preparation. M.E.B., data acquisition, data analysis, writing – original draft, and revisions. A.O., data acquisition. C.J.W., method development and writing – original draft. P.F.L., method development and writing – original draft. S.S., funding acquisition, supervision, and writing – original draft. D.A.P., conceptualization, data acquisition, data analysis, figure preparation, writing – original draft, and revisions. All authors critically reviewed the manuscript.

## Declaration of interests

S.S. is a consultant for Faron Pharmaceuticals and Simbec-Orion. C.J.W. is a consultant for Vantage Biosciences.
